# Plasticity in leukocyte migration during haematopoiesis and inflammation

**DOI:** 10.1007/s10974-025-09691-1

**Published:** 2025-02-18

**Authors:** C. Villella, M. Ciccioli, I. M. Anton, Y. Calle

**Affiliations:** 1 School of Life and Health Sciences, University of Roehampton, London, SW15 4JD UK; 2 Cellular and Molecular Department, Centro Nacional de Biotecnología (CNB-CSIC), 28049 Madrid, Spain

**Keywords:** Leukocytes, Migration, Plasticity, Adhesion, Cytoskeleton, Haematopoiesis, Inflammation

## Abstract

Under normal physiological conditions, leukocytes and other tissue resident immune cells have been shown to migrate using the mesenchymal (integrin/adhesion dependent) and/or ameboid (integrin/adhesion independent) modes of migration. The objective of this manuscript is to provide a comprehensive literature review that illustrates how leukocytes display high levels of plasticity shifting between ameboid to mesenchymal modes of migration during haematopoiesis and the inflammatory response. This plasticity is shaped by the reciprocal regulation between the pattern of gene expression associated with their haematopoietic lineage or the leukocyte activation status, and the response to the physicochemical and topological characteristics of the surrounding tissue. The use of some common elements from the F-actin polymerising and actomyosin machinery in both modes of migration may facilitate the high capacity of leukocytes to alternate between the two migration modes while navigating a highly heterogenous landscape of physicochemical cues in their anatomical journey. We discuss this paradigm using detailed examples of specific leukocyte populations such as dendritic cells, macrophages and lymphocytes. We propose that cell adhesions involved in leukocyte migration represent signalling hubs where differentiation and physicochemical cues converge. These molecular complexes then generate signalling outputs that coordinate leukocyte expansion, differentiation, and optimal patterns of cell migration during haematopoiesis and leukocyte recruitment to inflammation sites.

## Introduction

Cell migration plays a pivotal role in the function of immune cells, influencing their differentiation and response to inflammatory signals. During their lifespan, immune cells navigate tissues in different anatomical sites with highly divergent 2D and 3D topologies and physicochemical properties determined by the cellular and extracellular matrix (ECM) composition (Vargas et al. 2017). During haematopoiesis, migration of stem and progenitor cells across different functional regions in the bone marrow (BM) determines the generation of specific immune cell populations (Nagasawa 2006; Hassanshahi et al. 2019; Omatsu and Nagasawa 2021). Subsequently, partially or fully mature immune cells egress from the BM (Hassanshahi et al. 2019), reach the blood circulatory system and extravasate in secondary haematopoietic organs like the spleen, thymus or the lymph nodes to complete the differentiation process (Katakai and Kinashi 2016). Alternatively, if leaving the BM fully differentiated, they then exit the blood circulation to infiltrate target organs to patrol for detection of possible infectious organisms. In response to infections or tissue injury, circulating leukocytes are recruited to the insult sites to develop a fully built inflammatory response (Hassanshahi et al. 2019). All these processes require the adaptation of the migratory strategies of immune cells, using a range of versatile signalling mechanisms to respond to new tissue environments while adopting a differentiated or activated inflammatory phenotype.

This review focuses on the various roles of cell migration during haematopoiesis and the immune function of leukocytes. Additionally, it covers the current views regarding the modes of migration used by leukocytes in response to the topology and physicochemical properties of tissues. We will also discuss the possible signalling mechanisms involved in the interconnection between the processes of cell migration, and the differentiation and activation of leukocytes during the various steps of the inflammatory response.

## Cell migration modes and associated organisation of the cytoskeleton and cell adhesions in immune cells

### General cell migration modes.

Cell migration plays a pivotal role in numerous biological processes involved in embryonic development, normal physiology and disease. These include embryogenesis and tissue organisation and regeneration, the immune response as well as various aspects of wound healing (Kurosaka and Kashina 2008; Shaw and Martin 2016; SenGupta et al. 2021; Pourjafar and Tiwari 2024). Abnormal patterns of cell migration can result in pathological conditions such as cognitive impairment due to defects in neurological development (Borrell 2019), failure in healing resulting in chronical wounds (Peña and Martin 2024) and immune deficiencies (Rivers and Thrasher 2017). Additionally, the acquisition of a migratory capacity by cancer cells determines the progression of tumours towards aggressive phenotypes that facilitate the invasion into the local tissue and the colonisation of distant organs leading to formation of metastasis (Ghobrial 2012; Qu et al. 2023; Shi et al. 2024).

Michael Abercrombie firstly described the pattern of migration of individual metazoan cells in his pioneer studies by recording the movement of fibroblasts attached on a flat surface (Abercrombie et al. 1970, 1977; Abercrombie 1980). He defined a comprehensive four-step cycle of cell crawling on a 2D surface that explained how cells develop migration polarity with a protruding leading edge and a contracting rear end. In this model, the initial step of cell locomotion involves the protrusion of the cell membrane forming filopodia and lamellipodia followed by the formation of nascent adhesions that sustain the leading edge. Then, the adhesions mature in size while connecting with the actomyosin contractile machinery that pulls the cell body forward in the direction of the protruding end. In the final step, the rear adhesions are disassembled to facilitate the net translocation of the cell in the direction of movement. We now know that rather than occurring as isolated sequential events, these steps are integrated simultaneously and influence each other as the cells respond to the physicochemical features of their surroundings (Seetharaman and Etienne-Manneville 2020; SenGupta et al. 2021). In addition, since this first characterisation of cell movement, a more complex landscape of possible modes of migration in 2D and 3D environments has been described. However, in general, all the identified patterns of migration of animal cells share related mechanisms of force generation and transmission mediated by the organisation and dynamics of the actomyosin cytoskeleton (Yamada and Sixt 2019) coupled to various levels of adhesion and the rearrangements of the microtubule and intermediate filaments networks (Seetharaman and Etienne-Manneville 2020). Given the commonality in the signalling pathways involved in the different cell strategies for locomotion, cells may easily shift between modes of migration allowing for plasticity to adapt to changes encountered in their paths (Gandalovicova et al. 2016; Shaw and Martin 2016; Pourjafar and Tiwari 2024). The activation of a particular mechanism of migration depends on intrinsic factors such as the endogenous patterns of gene expression, and on the signalling pathways activated by environmental factors including biochemical (chemotactic molecules and adhesive ligands in the ECM or the surface of neighbouring cells), mechanical (rigidity and viscosity of the ECM) and geometrical cues (topology of the ECM) (Yamada and Sixt 2019; Merino-Casallo et al. 2022).

**Fig. 1:**
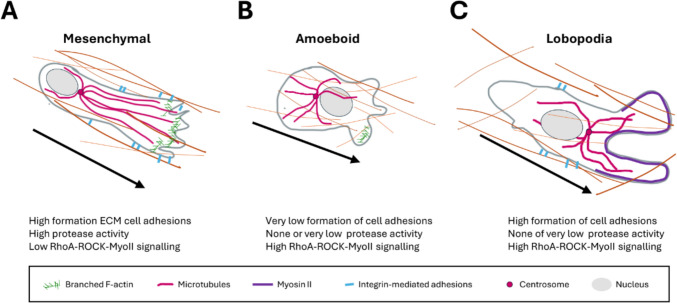
Single-cell modes of cell migration in a 3D environment. Schematic representation of the migration strategies used by single cells while moving in 3D environments. The black arrows show the direction of cell movement. The black arrows show the direction of cell movement. **A** Mesenchymal migration: cells acquire a characteristic spindle‐shaped morphology with protruding lamellipodia driven by the dynamic polymerisation of branched F-actin at the margin of the leading edge. The centrosome localises in front of the nucleus (between the nucleus and the leading edge) and organises the extension of the cytoskeletal networks towards the direction of movement; **B** Ameboid migration: cell shape is predominantly round with blebs generated by hydrostatic forces or by branched F-actin polymerisation at the leading edge. The centrosome is usually located behind the nucleus and cells form none or very low adhesive contacts with the surrounding ECM that lack proteolytic activity; and **C** Lobopodia-mediated migration: cells adopt an elongated shape and protrude using acto-myosin contractility at the leading front of the cell that pulls the nucleus forward like a piston creating hydrostatic pressure that results in the formation of large bleb-like protrusions named lobopodia. Cells form strong non-degradative adhesions and the centrosome localises in front of the nucleus

The pattern of cell movement described by Abercrombie is representative of the currently known as mesenchymal mode of cell migration (Fig. 1), typical of cells moving in 2D environments as well as certain cells migrating in 3D interstitial environments. In vivo 2D tissue structures include the membranes covering organs (the peritoneum, the lungs and thorax wall pleura), the ventricles of the brain as well as the inner surfaces of the endothelium in blood and lymph vessels (Friedl and Alexander 2011). However, most of the 2D surfaces in tissues, such as the bone surface or the basement membranes under epithelia or endothelia, are within a 3D context provided by a second opposing surface or a 3D scaffold of fibrillar ECM (Friedl and Alexander 2011), which renders cells migrating under confinement. Fibroblasts, various stem cells, immune and some cancer cells use mesenchymal migration in 2D and 3D environments. This mode of migration presents characteristic features such as: (a) the formation of a clearly structured network of F-actin at the protruding lamellipodia and filopodia; (b) the high dependence on formation of adhesions on the ECM or the surface of neighbouring cells that allow for the generation of strong traction forces through myosin-mediated contraction of F-actin networks; (c) the proteolytic capacity of these cell adhesions to degrade the ECM to facilitate cell migration; (d) the positioning of the centrosome between the nucleus and the leading edge; and (e) a clearly polarised and commonly elongated morphology (Raab and Discher 2017; Seetharaman and Etienne-Manneville 2020; SenGupta et al. 2021).

In 3D, cells may adopt mesenchymal, amoeboid or lobopodial modes of migration. Cells undergoing mesenchymal migration in 3D acquire a spindle‐shaped morphology and display multiple lamellipodia of smaller size than those developed in 2D that form at the tip of protruding membrane processes at the leading edge (Caswell and Zech 2018) (Fig. 1). The associated features of mesenchymal migration in 2D also apply to the 3D version of this model of migration (Caswell and Zech 2018; Yamada and Sixt 2019).

In contrast, cells using amoeboid migration exhibit a characteristic round morphology with a highly active cell surface due to formation of membrane blebs generated by hydrostatic pressure and/or by polymerisation of branched F-actin (Yamada and Sixt 2019; Seetharaman and Etienne-Manneville 2020). In ameboid cells, the centrosome is usually located behind the nucleus and cells form no or very low adhesive contacts with the surrounding ECM. Ameboid cells move in between the ECM fibres rather than degrade them to create a path. Although as a norm the adhesions of ameboid cells lack proteolytic activity, it has been reported that melanoma cells migrating in 3D environments show characteristic features of ameboid migration while secreting proteolytic enzymes that induce a diffuse degradation of the ECM (Orgaz et al. 2014).

Lastly, lobopodial migration in 3D is characteristic of individual cells that are tightly adhered on the surrounding matrix through strong adhesions that lack proteolytic activity. High levels of actomyosin contractility and nucleus movement forward in a piston-like manner create hydrostatic pressure at the front of the cell that results in the formation of a large form of bleb-like protrusions at the cell leading edge called lobopodia. The centrosome positions between the nucleus and the leading edge (Petrie et al. 2017; Seetharaman and Etienne-Manneville 2020; Pourjafar and Tiwari 2024).

Cells can also migrate collectively as cohesive groups of motile cells engaged to each other and the subjacent ECM through various possible types of adhesive contacts (Trepat et al. 2012). This type of migration involves the integration of external physicochemical cues across the cell mass, as well as inter-cellular mechanotransduction signals at cell–cell junctions (Ladoux and Mège 2017; Messer and McDonald 2023). Collective cell migration occurs during embryonic development and tissue differentiation, wound closure and is characteristic of some types of cancers (Pourjafar and Tiwari 2024).

This review focuses on the modes of cell migration identified in leukocytes and other immune cells, which only migrate individually using mesenchymal or amoeboid migration.

### Modes of migration and associated organisation of cell adhesions in immune cells

Leukocytes and other tissue resident immune cells have been shown to migrate using the mesenchymal and/or ameboid modes of migration. The type of migration strategy depends on the leukocyte type, the status of activation, and the topology and physicochemical properties of the surrounding microenvironment (Nourshargh and Alon 2014). Amoeboid movement is preferentially employed by leukocytes migrating in 3D environments containing fibrous ECM (Lämmermann et al. 2008). Bleb formation enables leukocyte migration through the ECM pores independently of the formation of integrin mediated adhesions (Lämmermann et al. 2008; Nourshargh and Alon 2014).

**Fig. 2:**
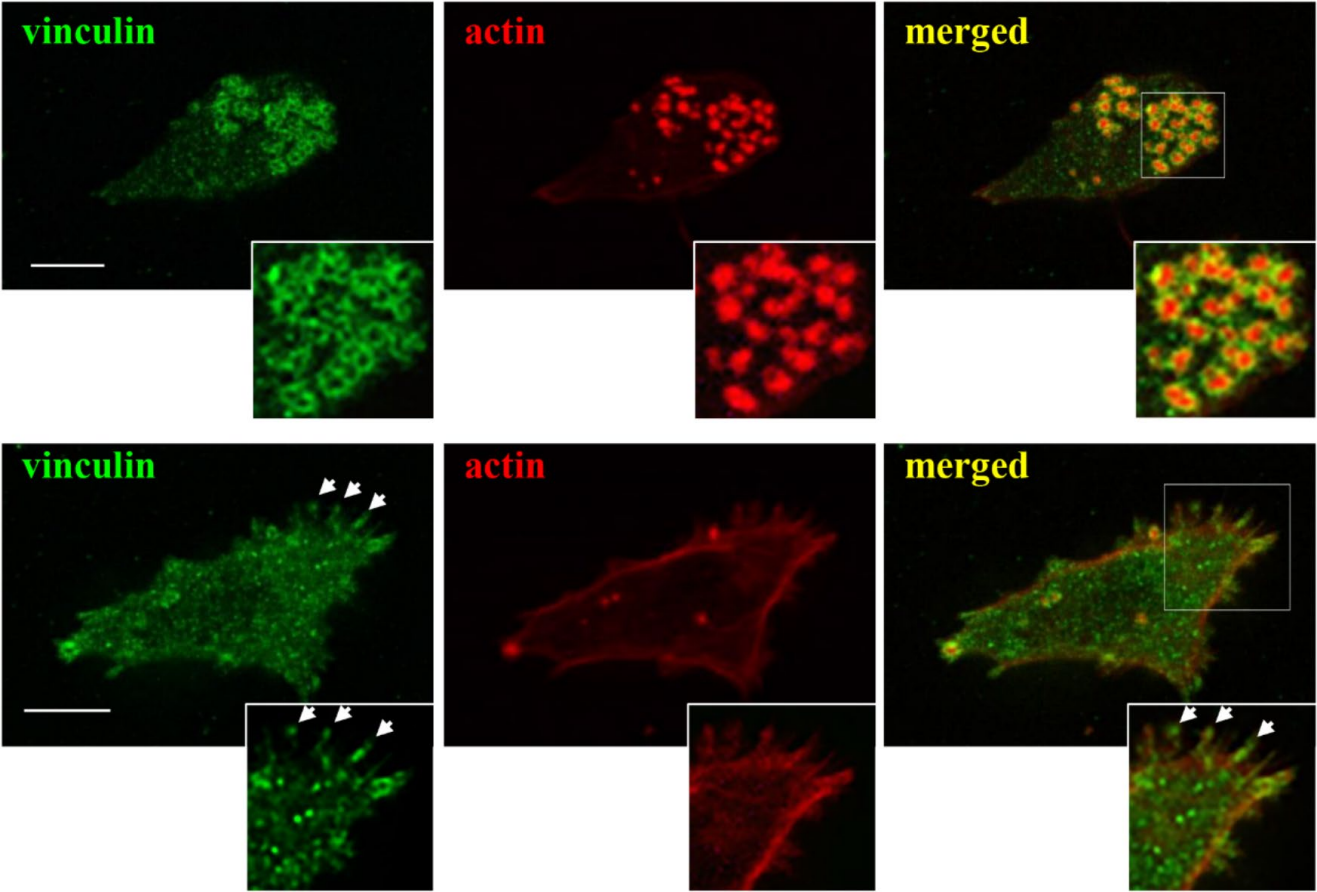
Formation of podosomes and focal adhesions in the THP-1 cells differentiated towards a macrophage phenotype. THP-1 cells were seeded overnight on 10 µg/ml fibronectin-coated coverslips in RPMI supplemented with 10% Foetal Calf Serum (FCS) and 1 ng/ml TGFβ1 for 16 h. Then, cells were maintained in RPMI supplemented with 10% FCS for additional 3 h (upper panels) or medium was replaced with RPMI devoid of FCS (lower panels). Cultures were then fixed with 4% paraformaldehyde, permeabilised with 0.05% Triton-X100 and immunostained to determine the distribution of F-actin (red) and the integrin-associated protein vinculin (green). THP-1 cells in complete medium formed clearly defined podosomes with a core of F-actin and a ring of vinculin (upper panels). Withdrawal of the FCS soluble factors from the culture medium resulted in loss of podosomes and THP-1 cells attaching to the substrate using focal complexes as shown by the clustering of vinculin forming punctuated or rod-shaped patches at the cell periphery (white arrow heads). We have previously shown that growth factors are required for podosomes initiation in a WASP/WIP-dependent manner (Griera et al. 2014). Magnifications of the boxed areas with vinculin, F-actin single staining and merged images are shown at the bottom or each micrograph. Bar 5 µm

Alternatively, leukocytes adopt mesenchymal migration in 2D and 3D environments and this involves the formation of integrin-mediated adhesions including focal contacts and podosomes (Fig. 2) (Calle et al. 2006; Cougoule et al. 2012). Focal contacts result from the clustering forming basal puncta of integrins and integrin-associated structural and regulatory proteins. Focal contacts can be, for example, identified in leukocytes crawling on the apical end of endothelial cells where these adhesions promote and sustain lamellipodium formation (Shulman et al. 2009; Alon and Shulman 2011). Assembly of focal contacts in leukocytes is induced by the presence of cytokines as well as cell surface receptors of endothelial cells such as ICAM-1 that binds β2 integrins (LFA-1) in crawling lymphocytes (Shulman et al. 2009).

**Fig. 3:**
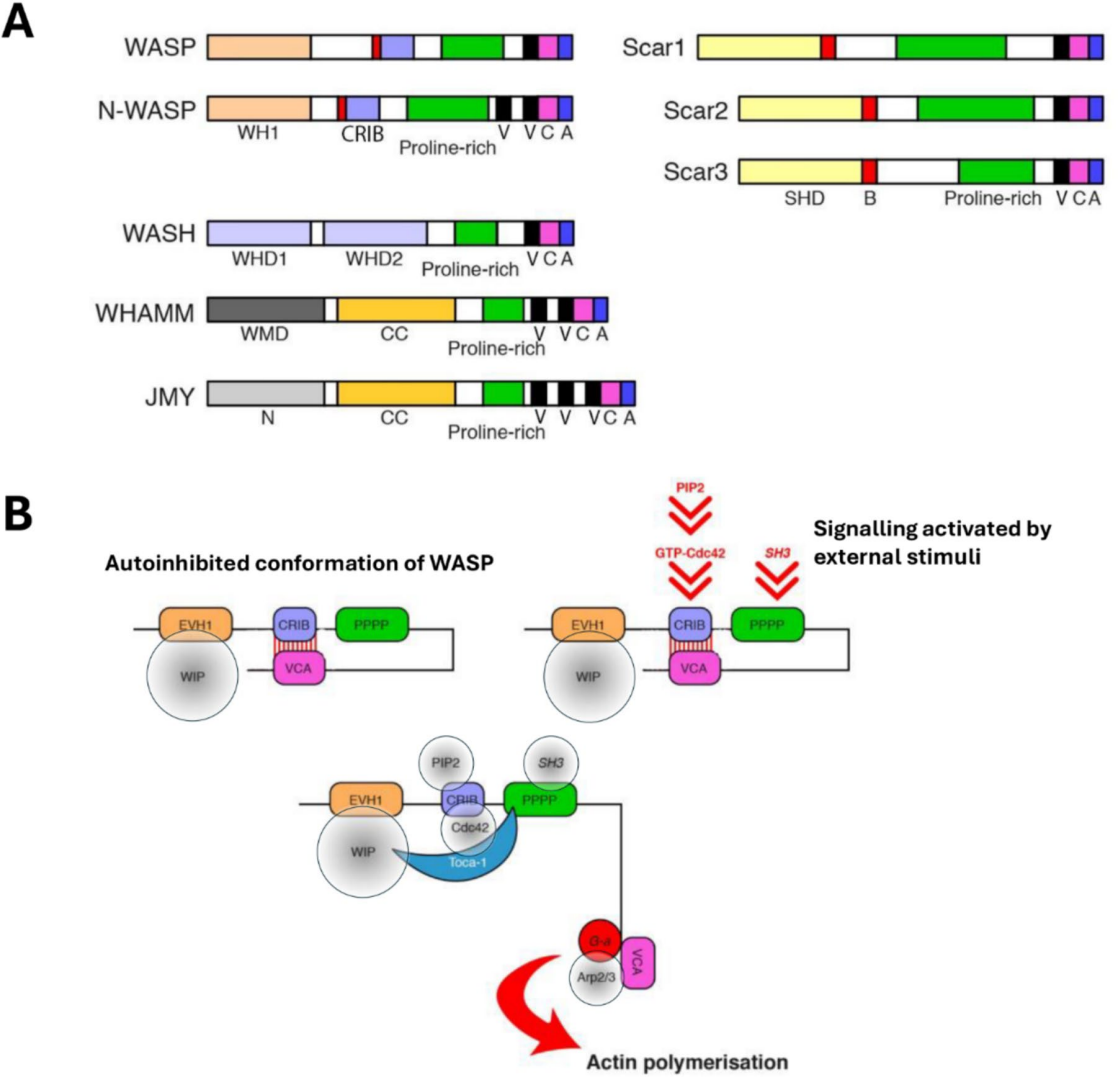
Schematic representation of WASP/Scar family of proteins and regulation of WASP activity.** A** Domain organization of WASP/Scar (also known as WASP/WAVE) family of proteins showing the major protein domains. All members of this protein family share a polyproline rich domain (Proline-rich), the Verprolin Cofilin Acidic domain (VCA) and a WASP homology 2 domain (WH2). The WASP subfamily proteins are characterized by a WASP homology 2 domain (WH1) in the amino-terminal region and a Cdc42 and Rac interactive binding (CRIB) domain. The Scar/WAVE subfamily of proteins lacks the CRIB domain and contains a characteristic SCAR/WAVE homology domain (SHD/WHD) and a basic domain (**B**). WASH comprises a WASH homology domain (WHD1) on the N-terminal and a tubulin binding domain (WHD2). WHAMM contains a membrane interaction domain (WMD) on the N-terminal and a coiled-coil (CC) microtubule binding domain, while JMY holds a coiled-coil domain at the N-terminal (N), a proline rich domain and on the C-terminal a VCA domain with three V sites. **B** In resting cells, WASP is found in its autoinhibited hairpin conformation facilitated by the interaction between the CRIB and the C-terminal domains while WIP interacts with the WH1 domain (red lines). Following external stimuli, several molecules get activated (red arrows represent the activation process). For example, Cdc42 acquires its GTP activated form, activation of PI3-kinase (PI3K) leads to the production of phosphatidylinositol 3,4,5-trisphosphate (PIP3) or some proteins, such as Nck, change conformation and expose SH3 domains that can bind the proline-rich (PPPP) domain in WASP. The Rho GTPase Cdc42 binds to the CRIB domain allowing the release of the VCA domain. The interaction with Cdc42, Toca-1, WIP and SH3-containing proteins with the proline-rich domain of WASP contributes to promoting the actin polymerization process (these bound proteins are represented by grey circles placed on the known binding domains of the open conformation of WASP). Once WASP is activated, the VCA region can interact with monomeric actin and the Arp2/3 complex and triggers the de novo actin polymerization

Podosomes are characteristic adhesions formed by leukocytes of the myeloid lineage including monocytes and monocyte-derived cells, neutrophils (Calle et al. 2006; Linder et al. 2023) as well as in megakaryocytes (Thomas et al. 2017; van den Dries et al. 2019) and endothelial cells during neovascularisation (Alonso et al. 2019, 2020). These adhesions display a characteristic organisation with a core of branched F-actin surrounded by a rim of integrins. The integrins in podosomes are coupled to many of the structural and signalling proteins commonly found in focal contacts and focal adhesions such as paxillin, talin and vinculin (Calle et al. 2006). Actin polymerisation in the core of leukocyte podosomes is driven by the Wiskott Aldrich Syndrome Protein (WASP), an adaptor protein that belongs to the larger WASP/Scar family of actin nucleation promoting factors (Alekhina et al. 2017; Rivers and Thrasher 2017), whose expression is restricted to haematopoietic cells under normal physiological conditions (Fig. 3) (Alekhina et al. 2017). WASP binds and activates the actin nucleating factor actin related protein 2/3 (Arp2/3) complex, which induces the polymerisation of branched F-actin at a ventral position close to the plasma membrane (Chou et al. 2006a; Monypenny et al. 2011; Alekhina et al. 2017). Podosomes are sites of proteolytic activity due to the focal secretion of matrix metalloproteinases (MMPs) and can mediate the degradation of the ECM as well as the activation of ECM-associated cytokines (Bañón-Rodríguez et al. 2011; Linder et al. 2023). Although all podosomes share this common structure, there are cell-specific variations in molecular composition and spatial organisation (Alonso et al. 2020; Linder et al. 2023).

## Role of cell migration in the biology of immune cells

Cell migration enables leukocytes to travel long distances over minutes or hours and reach different points within the same tissue or translocate to different organs. While navigating highly diverse environments, leukocytes integrate: chemical signals from cytokines, chemoattractants, cell adhesion molecules expressed on neighbouring cells and ECM ligands; mechanical signals produced by the particular rigidity and topology of the ECM; as well as signalling triggered by variations in temperature that emerge from the local or systemic hyperthermia produced during the inflammatory response (Simard et al. 2011). The capacity for interpretation of the surrounding signals and the subsequent type of migratory response largely depends on the gene expression profile of leukocytes associated with their haematopoietic lineage and the level of immune activation. Reciprocally, the mode of migration and the action of chemotactic cytokines can promote the differentiation and/or activation of immune cells to specialised phenotypes (Alraies et al. 2024).

The signalling pathways activated in leukocytes converge to produce a cytoskeletal and adhesion organisation that enables an optimal migratory strategy to navigate a specific environment. Given the frequent changes in the physicochemical properties of the microenvironment that leukocytes encounter, migration plasticity is critical to rapidly switch between different modes of migration to achieve the correct spatiotemporal localisation associated with their differentiation status and the inflammatory response. In this section, we review the currently proven patterns and modes of migration of immune cells during haematopoiesis and the inflammatory response.

### Leukocyte migration during haematopoiesis

Hematopoietic stem cells (HSCs) localise in the BM where they generate and maintain the lifelong output of blood and immune cells. The differentiation of the various lineages of leukocytes involves the migration of HSCs and haematopoietic progenitors through different BM functional niches as well as towards other peripheral germinal centres (Nagasawa 2006; Hassanshahi et al. 2019; Omatsu and Nagasawa 2021). The term “BM niches” refer to specialized regions in the bone cavity characterised by a specific cellular and ECM composition that create a local microenvironment with particular functionalities. HSC BM niches provide cellular and ECM adhesive surfaces, as well as secreted growth factors and cytokines that promote HSC survival, proliferation and differentiation in a tightly controlled manner. It is currently thought that the HSC BM niche is comprised of BM sinusoidal cells and associated perivascular cells that secrete high levels of C-X-C motif chemokine ligand 12 (CXCL12) also known as Stromal Derived Factor-1 (SDF-1) (Hassanshahi et al. 2019; Schürch et al. 2021; Omatsu and Nagasawa 2021). CXCL12/SDF-1 is a critical positional cue for the localisation and retention of HSC in the correct BM niche (Nagasawa 2015). It works as a chemoattractant by binding to the Gαi protein coupled receptor CXCR4 expressed in HSCs (Nagasawa 2006, 2015) and it is instrumental to induce HSC quiescence (Miao et al. 2020). Additionally, the dynamic organisation and the functionality of BM niches are regulated by the recruitment of mature leukocytes enabling the response to physiological requirements. Lymphocytes are key in this context given their role in regulating the inflammatory response and their migratory capacity. It has been hypothesized that the role of the pools of mature lymphocytes that enter and regulate the functionality of the BM (Broxmeyer et al. 2002; Libregts and Nolte 2014; Geerman et al. 2018) is to provide feedback related to the ongoing infection to mould a haematopoietic response adapted against the causing pathogen (Schürch et al. 2021).

**Fig. 4:**
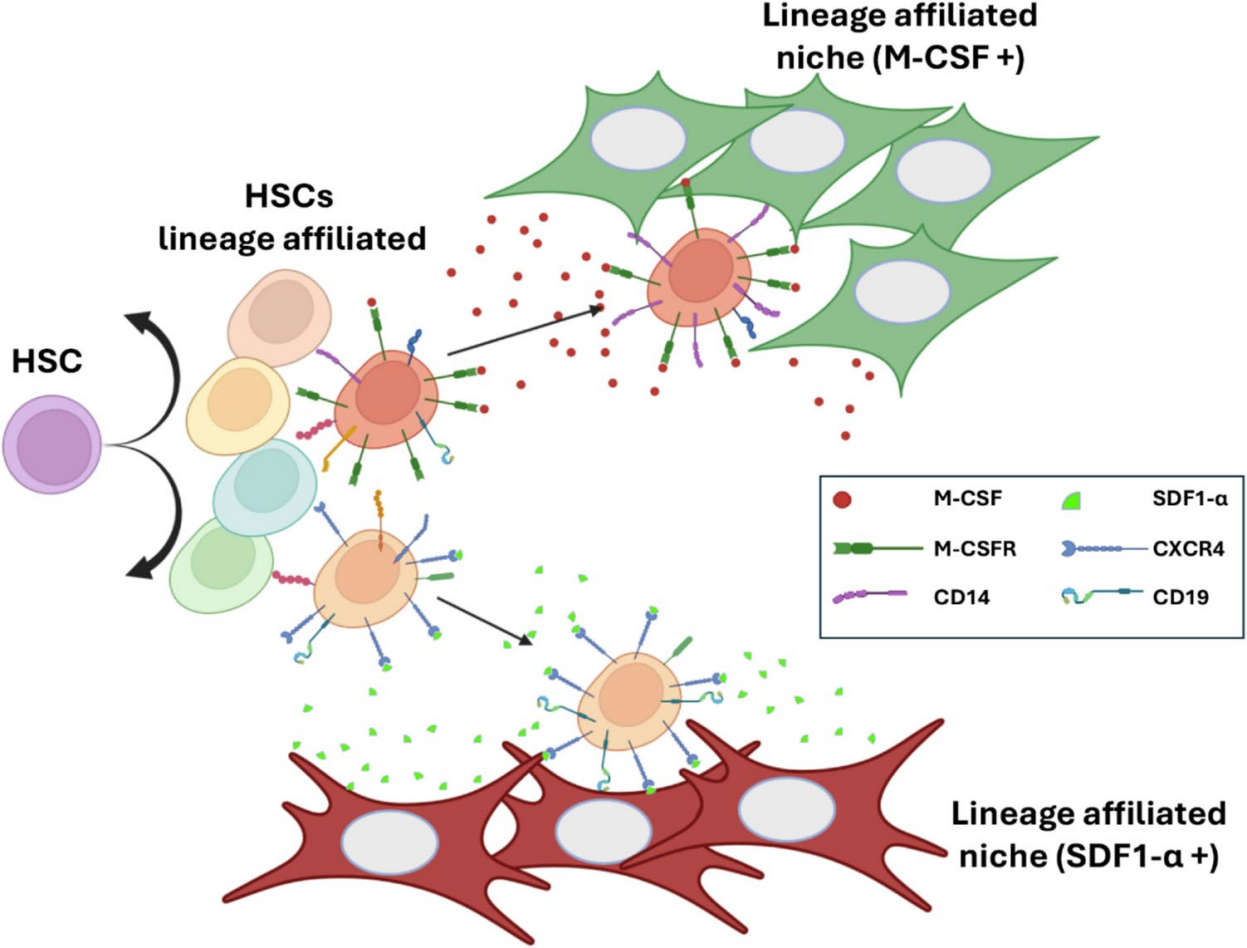
Expansion of haematopoietic lineages through natural selection of lineage affiliated HSCs. Proposed natural selection model of haematopoiesis where HSC undergo asymmetric cell division (bend black arrows) producing a new pluripotential HSC and one lineage affiliated HSC. Each lineage affiliated HSCs are committed towards a specific haematopoietic cell lineage by the low expression of the specific receptor for the lineage-inducing chemokine/cytokine that attracts the cell to the lineage differentiated lineage. For example, lineage committed HSCs expressing M-CSFR are attracted and migrate towards (linear black arrows) to the BM niche secreting M-CSF where these HSCs are stimulated to upregulate M-CSFR and other lineage specific receptors such as CD14. Similarly, HSCs expressing CXCR4 are attracted to the BM niche secreting SDF1α and are positively activated to upregulate CXCR4 and other lineage specific markers such as CD19. Further differentiation processes towards specific haematopoietic cell types of the same lineage would require a similar migratory process to additional BM differentiating niches leading to the generation of the various types of leukocytes. Figure created in BioRender. Anton, I. (2024) BioRender.com/f63v974

In order to trigger the process of haematopoiesis, exogenous signals in the HSC niche such as Stem Cell Factor induce the asymmetrical cell proliferation of HSCs, so that one of the daughter cells remains attached to the stem cell niche and the second cell is thought to acquire a lineage committed phenotype (Carpenter and Maryanovich 2024). It has been proposed that the lineage committed HSC expresses at low levels a restricted repertoire of chemokine receptors that facilitates the migration to a different BM niche (Brown 2020; Miao et al. 2020). Hence, pluripotent HSCs can generate a cohort of daughter lineage-committed HSCs that can be selected to migrate towards a large range of chemotactic and differentiating cytokines produced by the other BM niches (E.g. macrophage colony-stimulating, Granulocyte-monocyte stimulating factor) (Nagasawa 2006; Brown 2020). These BM niches reinforce the determination of a particular haematopoietic lineage by promoting the expression of specific receptors expressed initially at low levels by the recruited lineage committed HSCs (Fig. 4). The differentiating progenitors then expand in the new niches following a proposed model of natural selection (Yokota 2019; Brown 2020). Additionally, during the transition between BM niches, HSC encounter physicochemical signals that regulate cell migration while affecting their ability to self-renew and differentiate (Brown 2020). These include the level of rigidity of the plasma membrane and the expression of cell surface proteins in the neighbouring cells, the presence of cytokine and growth factors, as well as the composition and architecture of the ECM.

During these processes, immune cells need to coordinate cell migration across BM niches while acquiring a specific differentiation and/or functional maturation status and the feedback signalling between these activities is only partially understood. Furthermore, the specific adhesion and cytoskeletal remodelling or the modes of migration of immune cells across BM niches are largely unknown. To elucidate these processes, further studies of the dynamics of immune cells in the BM should combine functional assays with spatial proteomics and/or transcriptomics and 3D in vitro and intravital microscopy (Nombela-Arrieta and Manz 2017; Tikhonova et al. 2019; Baccin et al. 2020; Fooksman et al. 2024). This will enable a deeper understanding of the diversity, localization, and physiology of haematopoietic cells and will elucidate how they interact with the cellular components and the ECM within BM niches (Schürch et al. 2021). For example, recent intravital microscopy studies have shown that over 78% of mature plasma cells in the BM are motile (Benet et al. 2021; Aaron and Fooksman 2022) and this intrinsic motility changes upon their maturation state (Hauser et al. 2002; Fooksman et al. 2024). However, the cell migration mechanisms involved in the movement of plasma cells in the BM has not been yet characterised.

### Interdependence between the regulation of leukocyte migration and leukocyte differentiation: dendritic cells (DCs) as a representative model

Mature leukocytes released from the BM can undergo further processes of differentiation to become fully immunocompetent. Examples are the differentiation of T-cells into Th1 or Th2 helper phenotypes or the transition from an immature to a mature phenotype of DCs. In particular, the influence of cell migration in these differentiation or activation processes is well-documented in the maturation of DCs, which involves both mesenchymal and amoeboid migration modes.

DCs are professional antigen-presenting cells that link innate and adaptive immunity (Lipscomb and Masten 2002; Worbs et al. 2017; Patel et al. 2021). Their phagocytic function allows DCs to directly eliminate pathogens. At the same time, DCs can digest and present to T-cells the phagocyted pathogenic antigens to activate an adaptive immune response tailored to the particular infection. During homeostasis, DCs remain in an immature state probing the blood and tissues using pattern recognition receptors such as Toll-like receptors (TLRs) that bind to molecules shed or present on the surface of pathogens such as bacteria, viruses or fungi (Lipscomb and Masten 2002; Worbs et al. 2017; Patel et al. 2021). This steady state migratory behaviour of immature DCs while sampling their surrounding tissue has been demonstrated using intravital microscopy (Ng et al. 2008; Farache et al. 2013). TLRs binding to moieties in pathogens induce DC activation and development of a mature phenotype. Mature DCs are characterised by the upregulation of chemokine receptors such as CCR7, which recognises its ligands CCL19 and CCL21. These chemokines facilitate the recruitment of DCs to lymphatic vessels where they activate T cell responses through the upregulated expression, as part of the maturation process, of antigen-presenting and co-stimulatory proteins (Sozzani et al. 1998; Worbs et al. 2017; Cougoule et al. 2018).

Immature DCs have been shown to migrate using the mesenchymal mode in 2D (Calle et al. 2006; Alonso et al. 2019) and in 3D fibrous environments (Van Goethem et al. 2011; Cougoule et al. 2018; Alonso et al. 2019) facilitated by the adhesion through podosomes on the ECM. In 3D, DC podosomes localise at the tip of lamellipodia-driven long tubular protrusions (Cougoule et al. 2018) similarly to macrophages (Van Goethem et al. 2011). Moreover, the mesenchymal migration of immature DCs in 3D is dependent on the formation of podosomes rather than the maturation status as treatments that prevent podosome assembly, such as PGE_2,_ have been shown to induce ameboid migration in immature DCs embedded in fibrous environments (Cougoule et al. 2018).

Binding of TLR4 in DCs to bacterial lipopolysaccharides (LPS) activates the maturation of DCs in a process that results in podosome disassembly and acquisition of amoeboid migration in vivo and in in vitro 3D fibrous collagen models (van Helden et al. 2006; Lämmermann et al. 2008). In contrast, DCs matured upon TLR2 activation maintain the formation of podosomes in 3D and sustain the mesenchymal mode of migration (Cougoule et al. 2018). The motility modes resulting from the maturation status of DCs may be largely influenced by changes in the availability of the components of the F-actin polymerising machinery contributing to podosome dynamics and/or disassembly. Mesenchymal migration in immature DCs is regulated by two differentially regulated actin pools. At the leading edge, the activation of the Arp2/3 complex by signalling downstream of the Rho-GTPase Cdc42 controls the polymerisation of branched F-actin, which promotes a slower form of migration dependent on podosomes while promoting antigen capture. At the rear of DCs, the dynamics of the F-actin pool is regulated by the GTPase RhoA and the activation of the actin nucleation factor mDia1, which is central for cell contraction for forward movement coordinated with the protrusions at the leading edge. During TLR4 mediated DC maturation, the transition to the faster amoeboid migration correlates with a marked reduction of the Arp2/3 dependent actin polymerisation at the front while the mDia1-RhoA persists. This process promotes the amoeboid migration of LPS-matured DCs. Hence, the preferential use of actin nucleation factors during the maturation process of DCs determines the optimal mode migration required for the associated biological function (Vargas et al. 2016).

Taken together, these studies indicate that the type of stimuli inducing maturation determines the capacity of DCs to form podosomes. If podosomes are sustained, DCs undergo mesenchymal migration, whereas if podosomes are not formed, DCs move in 3D fibrous matrices using the amoeboid mode of migration (Cougoule et al. 2018). By decreasing the adhesion to the ECM, amoeboid migrating DCs attain faster locomotion (Alonso et al. 2019).

Reciprocally, the topology of the tissue microenvironment that DCs encounter in their migratory paths can induce the activation of a specific actin-polymerising machinery, which in turn may affect DC maturation. Hence, the physical properties of tissues can regulate the adaptive immune response by DCs (Alraies et al. 2024). During patrolling, immature DCs encounter a landscape of variable geometries that induce large shape changes in migrating DCs. Deformation of DC shape can induce the expression of maturation markers, which in turn promote the migration of DCs towards lymph nodes for antigen presentation to T-cells. At the molecular level, it has been shown that in response to nuclear tensioning resulting from physical constrains, DCs activate the WASP-Arp2/3 complex-mediated actin polymerisation in a process that depends on the lipid metabolism enzyme PLA_2_. As a result, DCs upregulate the expression of the mature DC activation marker CCR7 and activate the IKKβ–NF-κB-dependent pathway known to control the tolerogenic potential in mature DCs (Alraies et al. 2024). It is tempting to speculate that changes in the topology of the interstitial ECM due to physiological processes such as aging or pathological stresses like fibrotic responses may relate to the observed changes in immunocompetence of DCs (Lin et al. 2023; He et al. 2024). In summary, the current data indicate that DCs are very plastic and can easily switch between mesenchymal and amoeboid migration modes by integrating signalling induced by the type of maturation, and extracellular factors including the organisation, rigidity and composition of the ECM, as well as chemotactic factors (Cougoule et al. 2018; Alonso et al. 2019; Alraies et al. 2024).

### Regulation of leukocyte migration during the recruitment at sites of inflammation

Leukocyte migration has been predominantly studied in the context of their recruitment during inflammatory reactions in response to infection or injury (Wilgus 2008; Nourshargh and Alon 2014; Vestweber 2015; Rahman et al. 2021; Chesko and Wilgus 2022). Endothelial cells localised in the blood vessels around the area of infection or tissue damage become activated by the initiated inflammatory response and locally secrete chemotactic molecules in the luminal surface of the endothelium. This process triggers the recruitment of leukocytes from the blood circulation into the tissue subjacent to the activated endothelium by inducing the process known as the leukocyte adhesion cascade. Traditionally, this cascade is described in four consecutive interdependent steps: rolling, docking, diapedesis and invasion of the endothelial basal membrane by leukocytes. This adhesion cascade is applicable to lymphoid and myeloid leukocytes with variations in terms of the specific adhesive and migratory mechanisms as well as in the use of different modes of migration in the distinct steps of this process (Filippi 2019).

#### Rolling and docking

The first steps of the adhesion cascade encompass the initial tethering and rolling of circulating leukocytes on the apical surface of the activated endothelial cells followed by a firm integrin-mediated attachment (the docking), induced by the secreted and endothelial membrane associated chemokines such as IL-8, RANTES or MCP-1 (Patterson et al. 2002). Once tightly attached, leukocytes flatten and polarise to migrate attached on the apical surface of the endothelium to find permissive regions for diapedesis (transmigration across the endothelial barrier). During crawling on the apical surface of the endothelium, leukocytes form highly dynamic lamellipodia predominantly sustained by focal contacts that mediate the mesenchymal mode of migration (Shulman et al. 2009; Alon and Shulman 2011; Filippi 2019). This process involves the crosstalk between the different types of leukocytes and endothelial cells that results in specific changes in the physicochemical properties of the endothelial plasma membrane. As an example, the contact and migration of neutrophils on the apical surface of the endothelium increases the stiffness of the plasma membrane in endothelial cells (Wang et al. 2001) whereas monocyte adhesion and crawling decreases endothelial cell stiffness (Kataoka et al. 2002; Stroka and Aranda‐Espinoza 2010).

#### Diapedesis

Variations in the stiffness of the endothelial membrane induced by the attachment of leukocytes is a mechanical factor that may map specialised recognition areas potentially coupled with the pattern of transmigration of leukocyte types. For instance, the changes in endothelial cell stiffness induced by lymphocytes may be recognised and further activate the invasive protrusions used by this cell type to probe the endothelium. These invasive protrusions work as mechanosensors that explore the endothelial surfaces to identify regions with weak stiffness due to low actin density, or ineffective cell–cell junctions (Millán et al. 2006; Martinelli et al. 2014), which are permissive sites for transcellular and paracellular migration, respectively (Filippi 2019). In lymphocytes, invasive protrusions assembled during diapedesis have been related to the formation of various forms of adhesive strategies, including podosomes (Carman et al. 2007). To our knowledge, the factors that may trigger the preferred type of adhesion during diapedesis in lymphocytes and other leukocytes remain largely undefined. Similarly, the exact link between the possible mechanical mapping of the endothelial surface and the generation of the type of exploratory adhesions and membrane protrusions assembled by leukocytes has not been yet fully established.

#### Invasion of the basement membrane

To finally enter the inflamed tissue, leukocytes need to cross the basement membrane, a flat layer of crosslinked proteins located underneath of the endothelial cells (Filippi 2019). Basement membranes bear areas of different molecular composition and mechanical properties. The principal molecular components of basement membranes comprise laminins, collagen type IV, nidogens, and heparan sulfate proteoglycans (Korpos et al. 2010). Both the molecular composition and the stiffness of the basement membrane may determine the spatiotemporal recruitment of specific leukocyte populations by permitting and/or promoting particular modes of trans-basement membrane migration. For example, activated CD4^+^ lymphocytes transmigrate across specific basement membrane sites characterised by the presence of particular laminin isoforms; these differ from the laminin isoform composition of the crossing sites for macrophages or DCs (Korpos et al. 2010).

Both the mesenchymal (proteases-dependant) and amoeboid (protease-independent) modes of migration have been described during the transmigration of the basement membrane. For example, comparative migration studies in vivo showed that although both neutrophils and monocytes identify the same BM regions that exhibit low concentration of matrix proteins in the basal lamina, they use different migration strategies to get across. Neutrophils actively remodel and enlarge these basal lamina regions through the protease activity of elastase or MMPs (Reichel et al. 2008; Lin et al. 2008; Voisin et al. 2009) and hence, the degradation of laminin (but not type IV collagen) (Voisin et al. 2009; Nourshargh et al. 2010; Kelley et al. 2014). In contrast, monocytes have been shown to squeeze through similar regions without affecting the integrity of the basement membrane (Voisin et al. 2009). However, other studies suggest that cells of the myeloid lineage, including monocytes, DCs and macrophages, can use proteolytic-based mechanisms mediated by podosomes to degrade and trespass the basement membrane boundary during recruitment to tissues as well as during the egress of cells into the circulation (Calle et al. 2006; Holt et al. 2008; Kelley et al. 2014; Seano and Primo 2015; van den Dries et al. 2019; Bahr et al. 2022; Linder et al. 2023).

#### Interstitial migration of leukocytes

Once leukocytes trespass the endothelial basement membrane they need to penetrate the 3D interstitial space, which ranges in composition from a fibrous network of ECM to tissues formed by tightly packed cells (Nourshargh et al. 2010). The major components of the interstitial ECM include fibrillar collagen types I, III, and V, glycoproteins such as fibronectin, vitronectin, and tenascins, and dermatan sulfate and chondroitin sulfate proteoglycans (Korpos et al. 2010). Leukocytes have been shown to use amoeboid and/or mesenchymal modes of migration in this complex confined 3D environment, which may reflect the required plasticity in the migration strategy of leukocytes in the interstitium to successfully reach sites of inflammation. This plasticity may also allow DCs and macrophages to achieve a steady state of migration while patrolling tissues to detect the possible presence of pathogens. (Karrich et al. 2019). For example, monocytes, DCs, macrophages, neutrophils and T lymphocytes have been shown to migrate using amoeboid migration in fibrous ECM made up of polymerised collagen I (Cougoule et al. 2012; Vérollet et al. 2015a). However, when moving within fibrous and/or dense 3D environments, DCs and macrophages can also adopt a mesenchymal mode of migration using podosomes assembled behind lamellipodia. Similarly to 2D podosomes, 3D podosomes form rings of integrins and associated proteins surrounding a core of F-actin and they display proteolytic activity (Van Goethem et al. 2011; Alonso et al. 2019; Linder et al. 2023). However, the podosomes assembled in 3D gels containing collagen I fibres present a less distinct sharp conformation in comparison to those formed on flat surfaces. The shift between mesenchymal and amoeboid migration in macrophages in 3D has been reported to be regulated by the 3D topology of the ECM (fibrous *vs* dense) and the state of differentiation and/or activation (Cougoule et al. 2012). Specifically, Cougoule and colleagues showed that M2-polarized macrophages remodelled both fibrous and dense 3D matrices using podosomes, whereas M1 macrophages remained motionless in 2D and 3D ECMs. In the same study, peritoneal resident macrophages lacked podosomes and were only able to use amoeboid migration in fibrous ECM while thioglycolate-elicited peritoneal macrophages infiltrated fibrous and dense ECMs using podosomes (Cougoule et al. 2012). Similarly, activation of macrophages by exposure to HIV-1 virus inhibits their amoeboid mode of migration while promoting podosome formation and mesenchymal migration. This process involves the signalling mediated by the leukocyte-specific kinase Hck and WASP (Vérollet et al. 2015b).

Another example of plasticity in the mode of migration in interstitial spaces occurs during the recruitment of T-cells to peripheral lymph nodes. Extravasated T-cells in lymph nodes are thought to achieve high speed in the interstitial space by shifting between integrin-dependent and integrin-independent modes of migration depending on the density of the 3D ECM, and on the presence of fibroblastic reticular cells and DCs. Lysophosphatidic acid and chemokines secreted by fibroblastic reticular cells trigger T-cell migration and DCs facilitate anchorage-dependent motility via cell surface expression of ICAM-1 that binds to LFA-1 expressed by T-cells. These processes are integrated with the opposing mechanical signals induced by highly dense and confined ECMs that promote anchorage-independent motility. The resulting T-cell migratory strategy in these environments encompasses dynamic switches between migration modes that result in high-speed interstitial movement within the lymph nodes (Katakai and Kinashi 2016).

Taken together, the published work suggests that the integration of the signalling pathways activated in migrating leukocytes is cell type specific. The topology and molecular composition of the interstitial matrix together with the maturation and/or differentiation status of the leukocytes determine the favoured optimal migration strategy (mesenchymal and/or ameboid) that best facilitates the recruitment to sites of inflammation and the overall immune function.

## Leukocyte migration plasticity: potential role of WASP in the integration of cell migration and immune activation signalling in leukocytes?

The exact molecular mechanisms involved in coordinating leukocyte migration plasticity and the acquisition of a differentiation or immune competent phenotype remain largely uncharacterised. We propose that the signalling hubs that converge in cell adhesions and/or that regulate cytoskeletal remodelling in leukocytes are key in this context. Cell adhesion molecules and their intracellular partners have been shown to regulate gene expression and cell cycle progression in addition to their role in cell migration (Welsh et al. 2001; Klein et al. 2009). The connection between the organisation of integrin-mediated adhesions and cell cycle progression is further supported by the identified direct binding between the cell cycle regulator CDK1 and talin (Gough et al. 2021). Perhaps similar protein interactions at cell adhesions may be involved in the decision-making process of haematopoietic stem and progenitor cells needed to either sustain the undifferentiated phenotype and restricted proliferative capacity *vs* migrating and differentiate in the adequate BM niche or peripheral germinal centres. For example, talin is a key components of leukocyte podosomes and focal contacts (Calle et al. 2006). Whether talin binds CDK1 or other cell-cycle regulators at leukocyte adhesions and regulates cell cycle progression or differentiation has not been studied to date to our knowledge. Cell adhesions are also physically connected and regulate the acto-myosin, microtubules and intermediate filament cytoskeletal networks. The crosstalk between these cytoskeletal networks is involved in the development of migration modes within the mesenchymal/amoeboid spectrum (Seetharaman and Etienne-Manneville 2020; Kopf and Kiermaier 2021).

The protein WASP plays a pivotal role in cytoskeletal and adhesion dynamics in haematopoietic cells. In podosomes, WASP is localised in the core where it regulates actin and adhesion organisation and turnover (Monypenny et al. 2011; Macpherson et al. 2012). WASP also binds to microtubules through CIP4 (Tian et al. 2000) and the regulation of this complex by WASP has been shown to control microtubule organisation and podosome formation in myeloid cells (Linder et al. 2000). We have reported that WASP can associate with actin and vimentin in multiple myeloma cells (Ramasamy et al. 2017) further suggesting a possible role of WASP in the crosstalk between cytoskeletal networks in leukocytes. Additionally, some of the chemokines that activate WASP-mediated signalling in chemotactic processes, such as the response to macrophage-colony stimulating factor, also work as proliferative and differentiation signals (Pierce et al. 1990; Cammer et al. 2009). Therefore, WASP is a likely candidate that may coordinate the reciprocal crosstalk between the migration plasticity and differentiation/activation status in leukocytes during haematopoiesis and the inflammatory response (Rivers and Thrasher 2017).

The currently known functions of WASP in the nucleus as an epigenetic regulator (Sadhukhan et al. 2014; Sarkar et al. 2014; Chandnani et al. 2024) further suggest that this protein may be fundamental to integrate all these biological processes leading to the expansion of pools of immune cells while migrating to sites of inflammation or during haematopoiesis. The exact WASP-mediated molecular mechanisms involved in these processes are not completely understood.

It is also possible that WASP may coordinate the use of amoeboid and mesenchymal modes of migration in leukocytes traversing divergent 2D and 3D environments. WASP and the WASP interacting protein (WIP) work as a functional unit that regulates leukocyte polarity for migration, and podosome dynamics during the mesenchymal migration in leukocytes (Chou et al. 2006a; Monypenny et al. 2011; Vijayakumar et al. 2015). During podosome formation WASP and WIP control actin polymerisation in the core, the configuration and adhesion turnover of integrins in the rings (Chou et al. 2006b; Monypenny et al. 2011; Macpherson et al. 2012; Vijayakumar et al. 2015; Linder et al. 2023), and the recruitment of MMPs for matrix degradation (Bañón-Rodríguez et al. 2011). More recently, WASP has been involved in leukocyte amoeboid migration where cells push densely packed ECM matrix fibres or surrounding cells using WASP-induced actin patches reminiscent of podosome cores (these patches show protruding activity mediated by Arp2/3 complex-mediated polymerisation of branched F-actin) (Gaertner et al. 2022). However, these actin patches were devoid of a ring of integrins or any other cell adhesion molecules while they showed membrane-pushing activity induced by the mechanical load of the densely packed environment. Similarly to WASP, previous studies have involved WIP in the mesenchymal migration of leukocytes, particularly as a regulator of podosome formation (Chou et al. 2006b). However, in fibroblasts, WIP has been shown to regulate both mesenchymal and amoeboid migration modes (Banon-Rodriguez et al. 2013) raising the possibility that WIP may contribute to the shift between these two types of migration in leukocytes. Further studies may fully characterise the role of the WASP/WIP functional unit in the mesenchymal/ameboid plasticity of migrating leukocytes.

**Fig. 5:**
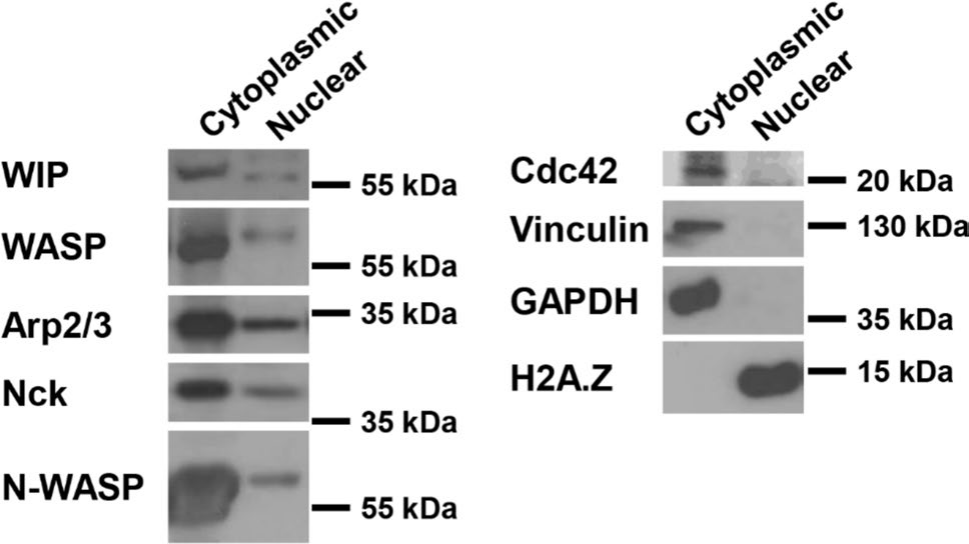
Detection of podosome components and actin regulators in the nucleus of the monocytic cell line THP-1. Identification by SDS-PAGE and immunoblot of various proteins in the biochemically separated nuclear and cytoplasmic fractions of THP-1 cells. THP-1 cells were seeded overnight on 10 µg/ml fibronectin-coated coverslips in RPMI culture medium supplemented with 10% FCS and 1 ng/ml TGFβ1 to induce differentiation towards a macrophage phenotype and podosome formation. We identified in the nuclear fraction of lysed cells as detected by western blot, the proteins WIP, WASP, Arp2/3, Nck and N-WASP. As we have previously shown, Cdc42 was detected only in the cellular cytoplasmic fraction (Ahmed et al. 2004). Proteins that are exclusively localised in the cytoplasm (vinculin and GAPDH) or the nucleus (histone H2A.Z) were used as internal quality controls of nuclear *vs* cytoplasmic fractionation

Importantly, WASP has also been shown to localise in the nucleus of T-cells (Sadhukhan et al. 2014; Sarkar et al. 2014, 2018). The nuclear activity of WASP regulates the differentiation of CD4^+^ T cells towards a Th1 phenotype by modulating histone modifications and the activity of RNA polymerase II, independently of its actin polymerising activity (Sadhukhan et al. 2014). In contrast, differentiation of Th2 cells is independent of the nuclear localisation of WASP (Sadhukhan et al. 2014). To our knowledge, whether the nuclear localisation of WASP may regulate the migratory capacity of T cells has not been determined yet. We have identified WASP in the nuclear fractions of myeloid cells (Fig. 5) where we also detected other podosomal constituents including Nck and p34-Arc/ARPC2 (one of the core components of the Arp2/3 complex) as well as the ubiquitously expressed member of the WASP/WAVE family of proteins N-WASP (Fig. 3). In contrast the Rho-GTPase Cdc42, which is a key activator of WASP function leading to actin polymerisation, was only detected in the cytoplasm (Fig. 5). WASP-mediated actin polymerisation downstream of Cdc42 in the cytoplasm may also influence gene expression. Formation of actin filaments in the cytoplasm reduces the availability of monomeric globular actin (G-actin), affecting the rate of actin polymerisation in the nucleus as well as the G-actin-dependent translocation of transcription factors such as myocardin-related transcription factor A (MRTF-A) (Davidson and Cadot 2021). Potentially, WASP could also regulate gene expression in immune cells by mediating cytoplasmic actin polymerisation around the nucleus. Perinuclear actin polymerization and actomyosin contraction regulate nuclear shape, which in turn influences chromatin accessibility, dynamics, and, consequently, gene expression. (Hu et al. 2019; Seirin-Lee et al. 2019; Davidson and Cadot 2021; Campellone et al. 2023). Future studies may clarify whether there is a nuclear-cytoplasmic interdependent regulation of WASP activity that may couple cell migration and differentiation of myeloid cells.

Several recent studies have shown that WASP is required for the expansion of particular leukocyte populations through its role regulating haematopoietic cell proliferation (Griera et al. 2014; Pereira et al. 2017; Biber et al. 2021; Vasconcelos-Fontes et al. 2024). Hence, lack of WASP results in reduced expansion of Tregs populations in vitro, correlating with the low number of these cells present in the thymus of WASP null mice (Vasconcelos-Fontes et al. 2024). The WASP-dependent molecular mechanisms involved in the regulation of cell proliferation remain unknown.

Nuclear WASP has also been shown to play a role in maintenance of DNA integrity in response to extracellular stress factors, as well as during cell division (Wen et al. 2020; Han et al. 2022; Nieminuszczy et al. 2023). This WASP function may be critical during the inflammatory response where leukocytes are embedded in a milieu with high concentrations of toxic molecules such as reactive oxygen species released by immune cells to kill pathogens. In this context, it is possible that WASP may protect DNA from damage while adjusting the optimal migration of leukocytes in inflamed tissues. Similarly, maintenance of DNA integrity is required for migrating cells in confined 3D ECM environments where nuclear deformation results in partial DNA damage (Kumar et al. 2014). Interestingly, we have also identified the presence of WIP in nuclear extracts of myeloid cells (Fig. 5). In fibroblasts, WIP also regulates the nuclear localisation of neural WASP (N-WASP) (the ubiquitous WASP homologous protein) (Vetterkind et al. 2002) raising the possibility that WIP may contribute to the nuclear function of WASP in immune cells.

In summary, the WASP/WIP functional unit plays critical cytoplasmic and nuclear roles, influencing leukocyte mesenchymal and amoeboid migration, gene expression, and the regulation of haematopoietic cell proliferation and differentiation. These proteins may comprise a molecular hub at immune cell adhesions, integrating differentiation and migration during haematopoiesis and leukocyte recruitment to inflammation sites. Further studies are needed to confirm this possibility.

## Conclusions and future directions

There is compelling evidence of the role of cell migration in haematopoiesis, immune activation and leukocyte recruitment to sites of inflammation. In these processes, leukocyte plasticity, characterized by the ability to shift between modes of mesenchymal and amoeboid migration, enables efficient locomotion through a wide variety of physicochemical, mechanical, and topological cues in 2D and 3D tissue environments. Overall, the current publications indicate that distinct patterns of migration plasticity are likely specific to individual immune cell types. The exact molecular mechanisms used by leukocytes to integrate these signals and shift between modes of migration while acquiring or maintaining specific forms of differentiation is poorly understood.

We propose that leukocyte adhesions comprise probing signalling hubs that get activated by the various migratory and differentiation physicochemical stimuli. The integrated signalling response emerging at adhesion sites may coordinate the efficient modes of migration, and leukocyte expansion and differentiation required for a competent immune response. Future studies on recently identified proteins involved in both mesenchymal and amoeboid migration and/or in other cellular functions such as proliferation or differentiation such as the WASP/WIP functional unit may shed light on the molecular mechanisms that orchestrate leukocyte migratory plasticity during haematopoiesis and the development of immunocompetence. 

## Data Availability

No datasets were generated or analysed during the current study.
